# Contextual Priors Shape Action Understanding before and beyond the Unfolding of Movement Kinematics

**DOI:** 10.3390/brainsci14020164

**Published:** 2024-02-06

**Authors:** Valentina Bianco, Alessandra Finisguerra, Cosimo Urgesi

**Affiliations:** 1 Department of Brain and Behavioural Sciences, University of Pavia, 27100 Pavia, Italy; valentina.bianco@unipv.it; 2 Laboratory of Cognitive Neuroscience, Department of Languages and Literatures, Communication, Education and Society, University of Udine, 33100 Udine, Italy; 3 Scientific Institute, IRCCS E. Medea, Pasian di Prato, 33037 Udine, Italy; alessandra.finisguerra@lanostrafamiglia.it

**Keywords:** action observation, action prediction, context, priors, predictive coding, autistic traits

## Abstract

Previous studies have shown that contextual information may aid in guessing the intention underlying others’ actions in conditions of perceptual ambiguity. Here, we aimed to evaluate the temporal deployment of contextual influence on action prediction with increasing availability of kinematic information during the observation of ongoing actions. We used action videos depicting an actor grasping an object placed on a container to perform individual or interpersonal actions featuring different kinematic profiles. Crucially, the container could be of different colors. First, in a familiarization phase, the probability of co-occurrence between each action kinematics and color cues was implicitly manipulated to 80% and 20%, thus generating contextual priors. Then, in a testing phase, participants were asked to predict action outcome when the same action videos were occluded at five different timeframes of the entire movement, ranging from when the actor was still to when the grasp of the object was fully accomplished. In this phase, all possible action–contextual cues’ associations were equally presented. The results showed that for all occlusion intervals, action prediction was more facilitated when action kinematics deployed in high- than low-probability contextual scenarios. Importantly, contextual priors shaped action prediction even in the latest occlusion intervals, where the kinematic cues clearly unveiled an action outcome that was previously associated with low-probability scenarios. These residual contextual effects were stronger in individuals with higher subclinical autistic traits. Our findings highlight the relative contribution of kinematic and contextual information to action understanding and provide evidence in favor of their continuous integration during action observation.

## 1. Introduction

Let us imagine observing a person inside a room approaching a semi-open door. What is he/she going to do: open or close the door? Apparently, the kinematics of the hand while reaching for the handle might be the same for both intentions; with this in mind, how can we properly guess the action unfolding and ultimately understand the underlying intention?

Relevant theories of action understanding, dominated by the feedforward recognition model [1], claim that we are able to infer other’s intentions by exploiting direct matching mechanisms between the observed actions and the motor acts stored in our motor system [2]. These processes are likely implemented by the action observation network (AON), a network including the inferior frontal gyrus (IFG), the adjacent premotor cortex, the rostral part of the inferior parietal lobule (IPL), and the superior temporal sulcus (STS) [2,3]. This view of a direct matching between observed actions and stored motor representations is supported by the notion that different action intentions are associated with different kinematics cues [4,5,6]. Indeed, during the observation of reach and grasp movements, the kinematics of the hand and digits approaching an object is differently engaged in anticipation of the upcoming intention. For instance, wrist height and grip aperture may discriminate between the actions of grasping a bottle to pour or to drink [7]. Empirical studies have shown that these kinematic signatures of action intentions are, at least partially, transparent to an observer, who is indeed able to discriminate between actions by capturing subtle kinematic cues that unveil intention [8,9]. Nevertheless, discrimination performance while observing movements in isolation remains far from perfect, being just above chance even for the relatively simple action of reaching to grasp an object like a bottle which offers limited intention alternatives [7,9]. This slight ability may hardly sustain our interactions in complex social environments. Further, there are several cases in which the available kinematic information is unintentionally obscured (e.g., occluded from view) or misleading (e.g., during active deception; [10]) regarding the ultimate intention of the actor, and the observer must therefore look at other sources of information to infer action intention.

Fortunately, in everyday life, actions do not occur in isolation but are always embedded in precise contextual scenarios, which prompt specific intentions. Therefore, when observing the actions performed by our peers, we may, even before the outset of the actual kinematics of the motor act, bias our predictions towards the most probable intention based on the potential affordance offered by the target object [11] and/or on the meaning of the context [12,13]. Going back to the initial question, we can guess if the person is going to open or close the door based on contextual cues. Maybe there is noise coming from the outside, prompting the action to close, or alternatively, it may be too hot in the room, prompting the action to open.

Predictive coding accounts of action comprehension [14,15] claim that inferring the intention behind an observed motor act is the result of prior expectations generated by previous experience, which are dynamically adjusted and updated based on prediction errors emerging from the actual sensory input. This view highlights the critical human ability of catching and learning statistical regularities of events in the surrounding environment (i.e., empirical Bayesian inference) and using this contextual information to aid both object recognition and action understanding [11,13,16,17]. At a neural level, the predicting coding model integrates the feedforward recognition model by claiming that all the AON areas are mutually interconnected to ensure that expectations of action intention can be promptly updated based on prediction errors [11]. Accordingly, a two-pathway model has been proposed. The encoding of action kinematics would involve the dorsal stream projecting from primary visual areas to parietal ones; conversely, information about the possible actions afforded by the target object would be conveyed through a ventral gradient from visual areas to the middle temporal gyrus and the anterior part of the inferior frontal gyrus. Crucially, the two-pathway model has been further expanded to include an additional stream that carries top-down predictions based on the contextual scenario, starting from fast projections from visual to prefrontal areas, which provide a coarse estimation of the possible action alternatives, and projecting back to frontal-parietal areas of the AON to disambiguate incoming kinematic information [11].

In keeping with the predictive coding account, Amoruso et al., [16] showed that perception of an action is actively affected by the embedding context. Indeed, action prediction was facilitated or interfered when movements kinematics unfolded within, respectively, congruent or incongruent contextual scenarios, as compared to an ambiguous one. This suggested that contextual expectations learned through previous experience help in disambiguating the meaning of observed actions in conditions of perceptual ambiguity [12].

In further studies [18,19,20,21], the associations between arbitrary contextual cues and specific actions (contextual priors) were formed from scratch by means of an implicit learning phase. In this phase, participants observed the full kinematics of an actor grasping an object (e.g., an apple) from a shallow holder (e.g., plate) with two possible intentions, namely individual (e.g., to eat) or interpersonal (e.g., to offer). In the implicit learning session, the occurrence of these action intentions was probabilistically associated with the color of the holder, thus forming contextual expectations. Afterwards, in a testing session, the same actions were presented, but video presentation was temporally occluded at the hand pre-shaping phase, when action kinematics was ambiguous and participants had to leverage previously learned contextual priors to guess the action intention. Collectively, the results showed that in the case of impoverished kinematic information, participants with neurotypical development, either adults [20,21] or children [19,22], were biased to the action intention suggested by contextual priors, with better performance when actions unfolded in highly associated contexts than in less-associated contexts.

Although these findings point to the critical role played by contextual expectations in aiding action understanding, it remains unclear whether contextual information serves action prediction only in conditions of perceptual ambiguity or it is continuously integrated with the unfolding kinematics to represent the ultimate action intention (and thus may also modulate action understanding in conditions of fully discernible kinematics). In keeping with the first account, it was shown that reliance on contextual cues is related to the reliability of kinematic information [9,23,24]. In particular, Koul et al. [24] found that when kinematic information was absent or scarce, action prediction was based on informative prior expectations; however, whenever kinematics provided sufficient cues for action discrimination, predictions were based on kinematics, irrespective of prior information. In a similar vein, a previous study [23] testing the modulation of the observer’s motor system during action observation showed that informative priors triggered motor activation even in the absence of reliable kinematic cues; however, compared to when only contextual information was available, motor facilitation was greater when both kinematics and priors were informative and converged into the same outcome prediction. Importantly, Cavallo and colleagues [25] also specified the crucial role played by timing-related information in leveraging contextual priors and action kinematics: at the beginning of a motor act, in the absence of relevant kinematic cues, the motor coding of the observed action mirrored the action goal mainly suggested by contextual scenario; later, as pertinent postural cues became available, motor activation resonated with the ongoing kinematics, irrespective of the contextual scenario. Therefore, the increased dependency on one source of information (i.e., action kinematics) or the other (i.e., contextual expectations) might be determined by the temporal evolution of the motor act, when kinematic cues progressively become more reliable, signaling specific action unfolding [26]. Conversely, according to predictive coding accounts [11,13], prior knowledge (i.e., contextual cues) and sensory inputs (i.e., action kinematics) are continuously integrated to ensure the best (and fastest) action recognition. Thus, contextual expectations should modulate action prediction at all stages of kinematics processing.

Of note, previous findings showed that individuals with autism spectrum disorder (ASD; [19]) failed to use contextual priors to predict unfolding kinematic information, showing an over-reliance on sensorial information at the expense of contextual experience-dependent expectations. Notably, there is a consensus in treating ASD as a dimensional spectrum, subtly blended with subclinical expressions within the general population [27,28]. In this vein, a significant correlation between autistic traits and the sensorial differences found in clinical ASD has been acknowledged by Grinter and co-authors [29]. They showed that compared to individuals with fewer autistic traits, individuals with more autistic traits have global processing difficulties similar to those often associated with clinical ASD. Autistic traits in the general population can be measured by a self-report questionnaire, the Autism Spectrum Quotient (AQ; [30]), which captures different domains related to social (i.e., social skills and communication) or non-social aspects of cognition (i.e., imagination, attention to detail, and attention switching). Notably, several previous studies assessing the ability to use prior contextual exposure to make predictions about upcoming experiences used the AQ [18,19,20,31]. Collectively, they all showed that high levels of autistic traits are associated with impairments in the integration of sensory evidence with contextual information.

To test whether and to what extent contextual modulation of action prediction is bound to the shortage of kinematic information, in the present study, we adapted the paradigm developed by Amoruso et al. [19] to obtain a testing phase in which videos were occluded at five different time intervals of the entire motor act. This allowed us to “snapshot” progressively distinctive kinematic information, ranging from when the actor was totally still to when the type of object grasping was fully discernible.

This experimental design aimed to disentangle two main alternative hypotheses: (i) if contextual priors are only used in case of uncertain kinematics cues, action prediction should be sensitive to them at early occlusion intervals, but it should be immune to them at later intervals, where the grasping of the hand is fully identifiable and action deciphering may fully rely on kinematic information; (ii) if contextual information is continuously weighted with kinematic information, beyond leveraging contextual cues when the kinematics of the hand is ambiguous, action prediction should be affected by prior context also at later intervals. In support of both hypotheses, we expected that for trials showing videos interrupted at early occlusion intervals, namely when the kinematics could not sufficiently distinguish between the two types of action, participants’ response would be fully biased towards the action intention suggested by contextual information. Instead, the two hypotheses diverge in terms of what concerns action prediction performance for later occlusion points, when the grasping provided enough kinematic information for proper action recognition: (i) the former would predict comparable performance for all trials, independent of the contextual association with high- or low-probability scenarios; meanwhile, (ii) the latter would predict a residual contextual effect.

Furthermore, since previous studies have suggested that a poor use of contextual priors in favor of the processing of sensorial information might be a hallmark of autistic traits [18,19,20,31], we also evaluated to what extent the ability to rely on contextual priors at different levels of perceptual uncertainty correlated with subclinical traits in a sample of neurotypical individuals. In line with these studies, we expected to detect lower use of contextual cues in individuals higher in subclinical autistic traits.

## 2. Materials and Methods

### 2.1. Participants

A total of 73 healthy young adults (48 F, mean age = 22.0, s.d. = 3.6 years, 3 left-handers) were recruited in the study. Participants were all university students recruited at the Faculty of Education Sciences at the University of Udine with a similar socioeconomic status. Inclusion criteria included age between 18 and 24 years, having normal or corrected-to-normal vision, absence of any neurological or psychological disorders, and not taking any psychotropic medications. The sample size was determined after testing the effects of a single predictor in the multiple regression analysis of the present study, where the total number of predictors (i.e., 5) corresponded to the subscales of the AQ. Using G*POWER 3.1 software [32], we estimated an expected effect size f(U) of 0.15 based on the predictive values of AQ traits in action–context integration in adults with typical development, setting the significance level at α = 0.05, and the desired power (1 − β) at 0.9. One participant was excluded for poor task adherence (a task accuracy of <0.65 in the familiarization phase).

The study received full approval from the local ethics committee (Comitato Etico Regionale Unico, Friuli Venezia Giulia, Italy, Parere CEUR-2021-Sper-65, 18 June 2021) and adhered to the ethical standards of the 1964 Declaration of Helsinki. All participants signed a written informed consent form and were informed of the hypothesis and aims of the study only after the end of the experimental sessions.

### 2.2. Stimuli

**Figure 1:**
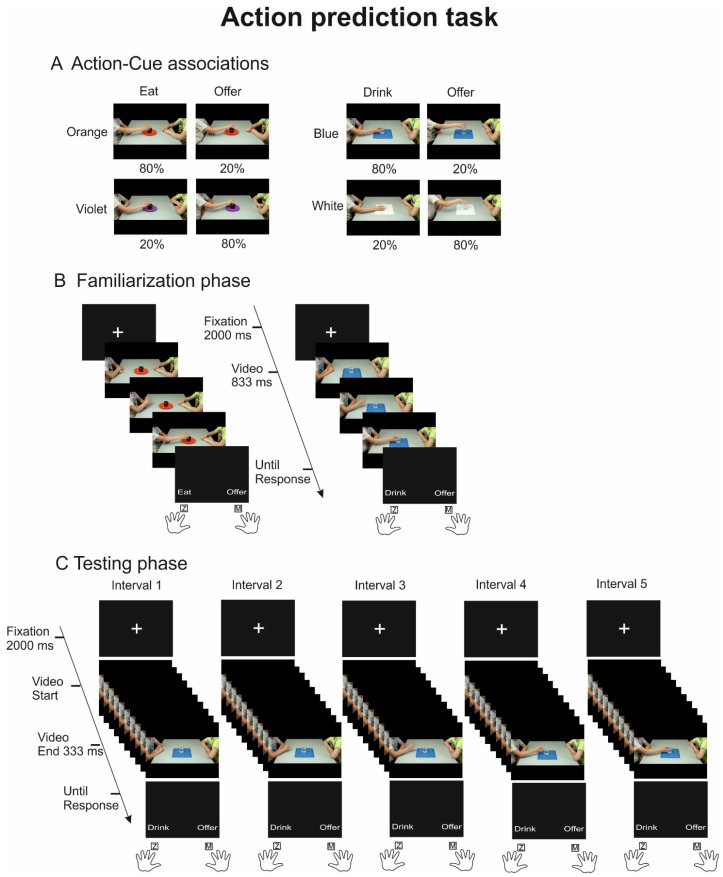
Action prediction task. (**A**) Example of probabilistic manipulations of action–cue associations for the eight videos used in the familiarization phase. For example, videos of the child grasping the apple positioned on an orange plate could be associated with the action of eating 80% of the time and with the action of offering 20% of the time. (**B**) Familiarization phase: in this phase, high and low probabilities of action–cue associations were created, videos lasted 833 ms, and kinematics was clearly identifiable, allowing a clear recognition of action intention. (**C**) Testing phase: in this stage, video durations were drastically reduced to 333 ms, capturing different time intervals of the videos previously used in the familiarization phase and interrupting the unfolding of the action at five occlusion intervals. Interval 1 covered the start of the video sequence when the child was still completely standing; interval 2 concerned the very beginning of the movement; interval 3 spanned the initial grasping movements of the hand; interval 4 started to truly unveil the intention of the grasp (e.g., drinking); and interval 5 showed the full reaching of the object, allowing a full recognition of the action intention. Time intervals from 1 to 5 progressively revealed action intentions thanks to the evolving kinematic information.

We used the same action-related videos employed by Amoruso et al., ([19], Figure 1A). The stimuli depicted a male child sitting at a table in front of a peer and reaching and grasping one of two different objects (a glass or an apple) using his right hand. The kinematics of the hand approaching the object prompted two different action intentions: a grip from the lateral side suggested an individual action (i.e., to drink or to eat), while a grip from the top provided the hint of an interpersonal action (i.e., to offer). The objects were presented with different arbitrary contextual cues: the glass could stand on a white- or a blue-colored tablecloth, while the apple could stand on an orange- or a violet-colored dish. In total, eight different videos were used. For more information on video recording and stimuli validation, please see [19].

### 2.3. Procedure

Considering the COVID-19 pandemic, participants performed the task remotely, after installing E-Prime 2 software subject session (Psychology Software Tools, Inc., Pittsburgh, PA, USA) on their own computers and assisted over the phone by the experimenter. The experiment consisted of two identical daily sessions separated by 2.7 ± 1.6 days. At the beginning of each experimental session, participants were instructed to find a room, sit in front of their computer screen (refresh rate 60 Hz) at a distance of 60 cm, and wear earphones. Stimulus size was adapted to cover an area of approximately 17° × 12° for all participants.

Before the beginning of the first session, participants received proper explanations over the phone. When providing task instructions, we particularly stressed the different ways of manipulating the objects used in the task (e.g., ‘when we grasp an apple with the intention to eat it, we usually approach it from the side’), but we did not provide any explicit clues regarding action–color association.

In each of two daily sessions, participants performed a familiarization phase followed by a testing phase (Figure 1). In both phases, the paradigm consisted of a two alternative force choice (2AFC) task: participants were instructed to observe the videos and to predict action intention (to drink/eat vs. to offer). In the familiarization phase, 25 frames of the videos were presented and the posture of the hand grasping the object from the side or from the top was clearly recognizable, thus allowing for straightforward action recognition. Of note, in this phase, the probability of co-occurrence of each action kinematics and color cue was implicitly manipulated to 80% and 20% (Figure 1A). To provide an example, trials showing the glass of water presented on a blue-colored tablecloth were associated with the intention to drink 80% of the time and with the intention to offer 20% of the time. Therefore, a trial showing a glass on a blue-colored tablecloth biased participants’ response towards choosing the option to drink. The familiarization phase of each session included two identical blocks of 80 trials each, with 64 trials associated with high prior and 24 to low prior probabilities. It is important to note that although identical probabilistic manipulations were maintained across all blocks of the familiarization phase for each participant, the action–cue probability associations were counterbalanced across participants.

In the testing session, all possible action–contextual cue associations were equally presented, but the duration of the video was limited to include only 10 frames extracted from the 25 frames of the same videos provided in the familiarization phase, thus capturing, and isolating different moments of action unfolding. Five different video time intervals were obtained for each video, ranging from the first interval (interval 1), when the child was completely still, to the last interval (interval 5), when the grasp was fully finalized. The intermediate intervals (2,3,4) progressively provided task-relevant grasp information, increasingly unveiling distinctive action kinematics (see kinematic analysis below). The testing phase of each session included two identical blocks of 120 trials each, and by listing the performance for the 2 objects associated with high and low probability priors in the 5 occlusion intervals, we obtained a total of 24 trials per conditions per session.

Each trial started with the appearance of a central fixation cross lasting 2000 ms, followed by one of the experimental videos, which was presented frame by frame at a rate of 30 Hz. In the familiarization phase (Figure 1B), videos lasted 833 ms (25 frames), while in the testing phase (Figure 1C), videos lasted 333 ms (10 frames). At the end of each video, a response prompt appeared, providing the two verbal descriptors associated with the response options (to eat/drink, to offer) at the bottom left and right of the screen. The response was provided by pressing a key using the index fingers; the (Z) or (M) keys on the keyboard corresponded to the left and right location of the descriptors. Response speed and accuracy were equally encouraged. The prompt disappeared after participant’s response, followed by an empty black screen lasting 1000 ms before the start of the upcoming trial.

At the end of the second session, participants completed the Autism Spectrum Quotient (AQ, [30]). The AQ comprises 50 questions, made up of 10 items assessing the extent of autistic traits in 5 different areas: social skills, imagination, communication, attention switching and attention to details. For each question, participants must indicate the extent to which they concur with the statement provided, by means of a 4-point Likert-like scale: 1 = “definitely agree”, 2 = “slightly agree”, 3 = “slightly disagree”, and 4 = “definitely disagree”. The range of scores for each subscale is from 0 to 10, with higher scores suggesting higher presence of autistic traits for the different domains.

### 2.4. Stimulus Kinematic Analysis

**Figure 2:**
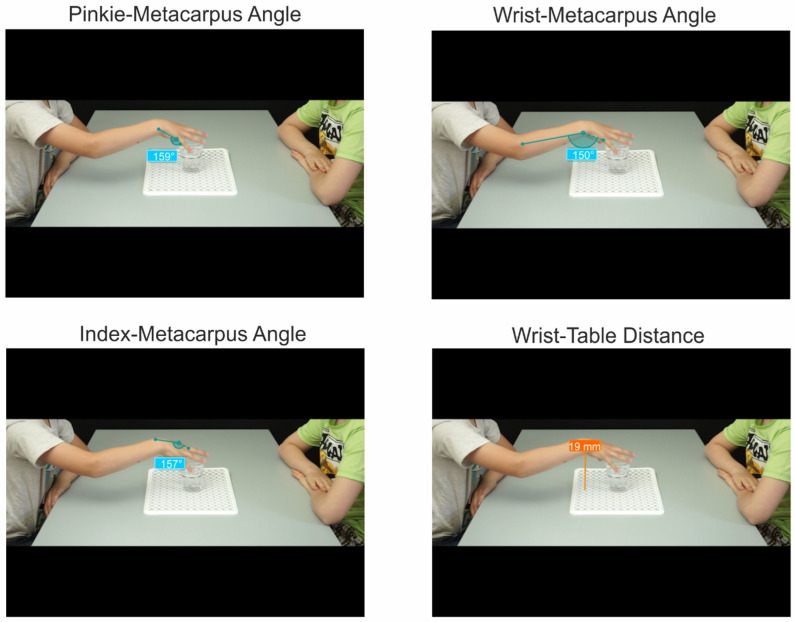
Examples of kinematic parameters extracted from the last frame of Interval 5 of the videos showing distinctive kinematics associated with the action “to offer”.

Since the main purpose of the study was to test to what extent the contextual cues modulated action recognition with progressively increasing kinematic information at different moments of action unfolding, we performed a kinematic analysis of stimuli. To detect the similarities/differences in the kinematics profile for each grasp-and-reach action, we used Kinovea software (Version 0.8.15) to extract distinctive spatial parameters from the last video frame (i.e., the 10th) of each interval for each action. The visible joint angles were calculated in degrees as follows. The pinkie–metacarpus angle was extracted from the line connecting the proximal–middle phalange joint with the proximal phalange–metacarpus joint of the pinkie and the line connecting the latter with the ulnar styloid process. The wrist–metacarpus angle was defined by the line connecting proximal phalange–metacarpus joint of the pinkie with the ulnar styloid process and the line connecting the latter with the lateral epicondyle. The index–metacarpus angle was extracted from the line connecting the proximal–middle phalange joint of the index with the metacarpus–proximal phalange joint and the latter with the radial styloid process. Additionally, the wrist–table distance was measured in mm as the perpendicular line joining the ulnar styloid process and the surface of the table. Figure 2 shows an example of the kinematic parameters extracted in one of the experimental conditions.

**Figure 3:**
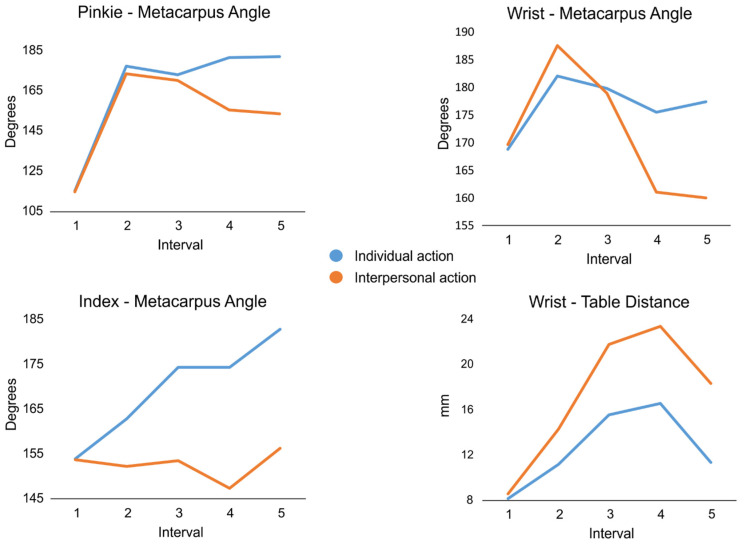
Line plots of the kinematic parameters for each action (individual vs. interpersonal) in the five time intervals. Top left: pinkie–metacarpus angle (in degrees); top right: wrist–metacarpus angle (in degrees); bottom left: index–metacarpus angle (in degrees); bottom right: wrist–table distance (in millimeters).

The extracted values for the selected frames are shown in Figure 3. The qualitative observation of the line plots collectively showed increasingly different kinematic profiles for the individual (i.e., eat/drink) vs. interpersonal (i.e., offer) actions. Specifically, occlusion interval 1 showed the child still, and we expected that participants’ response should be fully biased towards the action associated with the highest prior probability, as learnt in the familiarization phase. Likewise, occlusion interval 2, by showing the very beginning of the movement, would not provide enough information for proper action prediction and would encourage the participant again to opt for the action hinted by the contextual cue associated with the highest probability of co-occurrence. Later, occlusion interval 3 provided initial differences in action kinematics, followed by occlusion intervals 4 and 5, where these differences became increasingly evident. Therefore, we expected that participants should be progressively more able to catch kinematics distinctions, showing comparable action recognition performance between trials associated with high and low probabilities of action–cue co-occurrence.

### 2.5. Data Handling and Analysis

Data from the two sessions were collapsed. For the familiarization phase, we averaged response accuracy between the probability conditions (i.e., 20%, 80%) separately for the individual and the interpersonal actions. Participants that did not reach a response accuracy of at least 65% at this stage (one participant) were excluded from further analysis.

For the testing phase, we analyzed and assessed participants’ performance by adopting signal detection theory [33]. We treated videos showing individual and interpersonal actions as separate categories; correct responses for the former were considered hits, while erroneous responses to the latter (i.e., interpersonal actions identified as individual) were classified as false alarms. We obtained bias-corrected measures of sensitivity in discriminating between the two options (i.e., d’) by transforming the response ratio to *z*-scores, and then subtracting the *z*-score that corresponds to the false alarm rate from the *z*-score that corresponds to the hit rate [33]. Additionally, an index of response bias (i.e., criterion, c) was calculated for each experimental condition by averaging the z-score corresponding to the hit rate and the z-score corresponding to the false alarm rate and then multiplying the result by −1 [33]. The d’ and c values obtained for high vs. low prior probabilities for the five occlusion intervals were subjected to a 2 × 5 repeated-measures analysis of variance (RM-ANOVA) with prior probability (high vs. low) and occlusion interval (1 vs. 2 vs. 3 vs. 4 vs. 5) as within-subjects variables. Additionally, estimates of the effect size were reported using the partial η squared (η_p_^2^) [34]. Post hoc analyses were carried out using Duncan’s test to correct for multiple comparisons and explore significant interaction effects. This sequential post hoc test reduces the size of the critical difference depending on the number of steps separating the ordered means and is optimal for testing in the same design effects that may have different sizes [35,36,37].

Further, based on the results of the RM-ANOVA, we were interested in evaluating if the reliance on the contextual prior in the first occlusion interval, where the kinematics information was fully uninformative, and in the last occlusion interval, where the kinematics information was fully discernible, was related to individual levels of autistic traits. In this regard, previous studies have not addressed if the use of prior probability in the case of information dependent fully and only on context (interval 1) or on unambiguous kinematics (interval 5) is related to individual autistic traits. Therefore, for the first and last occlusion intervals of the action prediction performance of each participant, we calculated a contextual facilitation index by subtracting the d’ value obtained in the condition of lower probability (20%) from that obtained in the condition of higher probability (80%). Considering previous evidence of an association between AQ scores and contextual prior effects [20,21,31], these indices were then entered into multiple linear regression analyses together with the five AQ subscales as selected predictors.

For the regression models, we used standard procedures for assessing multicollinearity and ensuring that tolerance was not lower than 0.4.

All analyses were performed in Statistica 10 software (Statsoft, Tulsa, OK, USA). The alpha value for all statistical tests was set at 0.05.

## 3. Results

**Figure 4:**
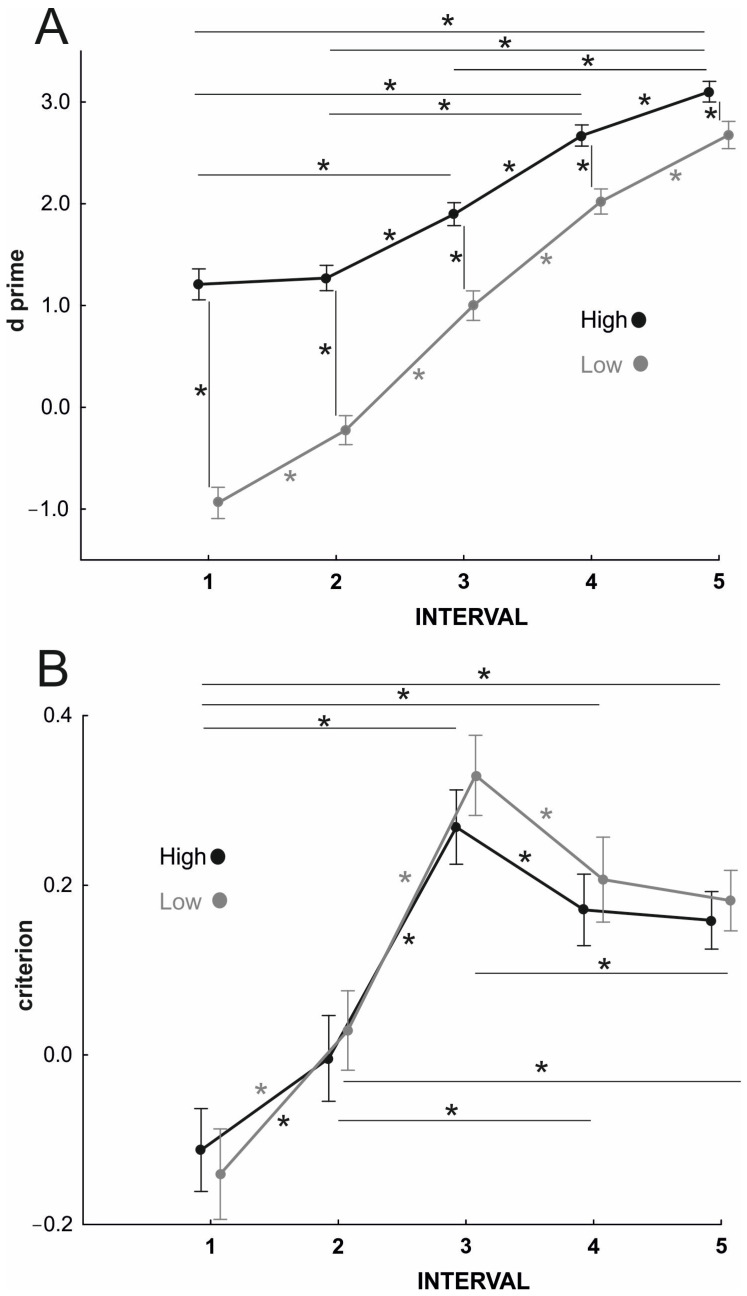
(**A**) Behavioral performance of action prediction associated with high and low prior probabilities for the five occlusion intervals expressed as d’; (**B**) Participants’ response biases of action prediction performance associated with high and low prior probabilities for the five occlusion intervals, expressed as c. Asterisks indicate significant comparisons (*p* < 0.05) between consecutive intervals and between high and low prior probabilities within each interval. Error bars represent SEM.

The RM-ANOVA on the d’ values yielded significant effects of prior probability (F_1,71_ = 44.10, *p* < 0.001, η_p_^2^ = 0.38), interval (F_4,284_ = 387.55, *p* < 0.001, η_p_^2^ = 0.84) and prior probability × interval interaction (F_4,284_ = 31.10, *p* < 0.001, η_p_^2^ = 0.30). Post hoc analysis revealed that, for all intervals, prediction performance was better for high prior probabilities than for low prior probabilities at all occlusion intervals (all *p*s < 0.001, Figure 4). Furthermore, performance improved across longer occlusion intervals for both high (all *p*s < 0.001) and low prior trials (all *p*s < 0.001), except between interval 1 and interval 2, where a significant improvement emerged only for low (*p* < 0.001) but not for high prior trials (*p* = 0.619). Overall, these effects suggest that despite the observed differences in the evolving kinematics between individual and interpersonal actions, participants were still integrating kinematics with contextual information even when kinematic unfolding was clear. Furthermore, when contextual priors were highly informative, no performance advantage was provided by the kinematic information unfolding in initial phases of action deployment.

The RM-ANOVA on the c values only yielded a significant main effect for occlusion interval (F_4,284_ = 30.79, *p* < 0.001, η_p_^2^ = 0.30). Post hoc analysis showed significant differences among all intervals (all *p*s < 0.009) except for the comparison between interval 4 and interval 5 (*p* = 0.663). The effects of prior probability (F_1,71_ = 0.91, *p* = 0.344, η_p_^2^ = 0.13) and prior probability x occlusion interval interaction (F_4,284_ = 0.49, *p* = 0.742, η_p_^2^ = 0.01) were not significant. Collectively, these findings suggest that despite a response bias in reporting an interindividual action emerging along the initial action deployment, this was not modulated by the contextual information provided by prior probability.

### Multiple Linear Regression Analyses

The individual AQ total scores ranged between 6 and 29 (Mean = 17.6, s.d. = 5.7), spanning from low (i.e., <13) to high (i.e., >18) levels of autistic traits [30]. See Table 1 for subscale scores.

**Figure 5:**
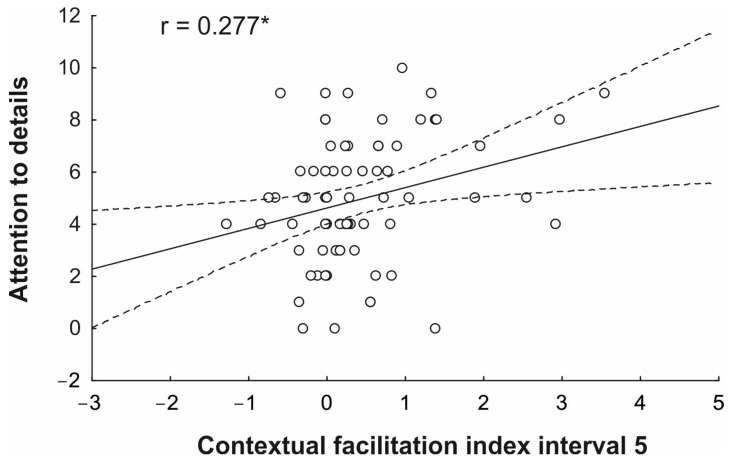
Correlational results showing a significant positive correlation between the contextual facilitation index for interval 5 and attention to detail scores. The asterisk signals the presence of a significant correlation.

The summary statistics of standard multiple regression analyses conducted separately for the Contextual Facilitation Index of interval 1 and interval 5 are reported in Table 2. Multi-collinearity statistics confirmed that the assumption was not violated (tolerance > 0.4). For the Contextual Facilitation Index associated with interval 1, none of the subscale scores were a significant predictor (whole model: adjusted R^2^ = 0.034; F_5,66_ = 0.47; *p* = 0.798), suggesting that prior context was guiding action prediction independently of any individual autistic traits. Conversely, for the Contextual Facilitation Index at interval 5, the attention to detail subscale score was a significant predictor (*p* = 0.022; whole model: adjusted R^2^ = 0.087; F_5,66_ = 2.347; *p* = 0.05), while the other subscale scores were not reliable predictors (all *p* > 0.134). This result points at the existence of a positive correlation between cognition and autistic traits, reflecting an increased attention to detail and the ability to leverage contextual information even when kinematic information is more informative. This significant correlation is further illustrated in Figure 5.

## 4. Discussion

The aim of the present study was to investigate the relative weight of kinematics and prior contextual information during the understanding of actions. More precisely, we tested two main hypotheses: (i) that the role of context is mainly to “disambiguate” the kinematics in cases of perceptual uncertainty, and (ii) that context and kinematics information are continuously integrated independently of the availability of perceptual information.

We used an action prediction task in which we concurrently manipulated the extent of available kinematics information and the strength of association between actions and contextual cues. First, participants underwent a familiarization phase consisting of full-length action videos with clearly identifiable kinematics wherein contextual associations were formed, generating high- and low-probability action–cue scenarios. Afterwards, in a testing phase, the original videos were cut into shorter videos of equal duration and limited to five different timeframes of the complete action act. We expected that occluding the videos at earlier time intervals, in which scarce kinematics information was provided, would encourage prediction in favor of the action associated with highly informative contextual priors; conversely, findings obtained from the later occlusion intervals, in which unequivocal kinematics cues were given, should provide crucial evidence for the extent of integration between sensorial and contextual information during action recognition.

Overall, our findings showed that for earlier timeframes, participants’ responses were biased in favor of the action associated with the high-probability action–cue scenarios; importantly, even for later timeframes, despite the increased availability of informative kinematics cues, action prediction was still biased in favor of the intention suggested by the prior context. This latter finding is particularly relevant and provides evidence supporting a continuous integration between contextual information and movement information even in the presence of fully discernible kinematics (hypothesis 2).

These findings relate to Bayesian views of understanding action [14], which posit that our brain actively and continuously combines the expectations generated by prior context with incoming sensorial information. Prominent scientific evidence has demonstrated that action recognition does not depend solely on action kinematics but also on the presence of the objects in the scenario which elicit distinctive action affordances, thus generating expectations (i.e., priors) even before the beginning of the action. In this regard, Iacoboni et al. [38], showed that premotor areas involved in action recognition are also involved in action understanding, pioneering the crucial role played by the context in clarifying the intentions behind the actions of others; indeed, observing the same action performed in different contexts prompt different meanings, thus reflecting different intentions. According to these accounts, we perceive a “contextualized kinematics” in which prior knowledge aids action recognition by suggesting the intentions that are more probably associated with a distinctive motor act in a specific context [12,15].

The observed reliance on contextual prior information at the first occlusion points (time intervals 1 and 2), where informative kinematics information was hardly absent, reconcile with prominent transcranial magnetic stimulation (TMS) studies claiming that our motor system mainly resonates with the action goal [39] depending on the availability and type of information at hand in a specific processing stage [24]. Accordingly, at the beginning of the motor act, given the lack of informative kinematic cues, the possible action intentions are almost entirely derived from implicitly learnt, higher-order contextual action–cue associations. Therefore, contextual information is effectively exploited during action observation, when visual information about the action itself is absent. Presumably, at this stage, the action perception activity of the AON would most likely code the high-level intention of the observed contextualized motor act. The context-based expectations of the goal of the action generated in prefrontal areas are sent backward to posterior areas without accounting for any prediction errors; this is due to the lack of any motor/kinematics cues.

With evolving kinematic information, at the intermediate occlusion point (interval 3), although kinematics cues revealed increasingly perceivable pre-shaping features, they remained particularly ambiguous. In line with previous investigations using similar experimental stimuli and interrupting videos at the same occlusion point as the last video frame of interval 3, we further confirmed that under conditions of dubious kinematic information, previous exposure to arbitrary action–cue associations induces action prediction biases in favor of the action associated with the highest-probability scenarios [16,17,20,21]. Importantly, these previous studies manipulated the availability of kinematic information by showing increasingly longer videos (see also [8]). This way, they concurrently increased not only the availability of kinematic information but also the timing of contextual processing, since context was presented for longer. Amoruso and co-authors [16] showed that improvement in prediction performance for congruent contexts and its impairment for incongruent contexts affected action prediction with different timings. Namely, a selective facilitation of performance occurred for actions embedded in congruent contexts only at early stages of processing, while a selective inhibition occurred for actions embedded in incongruent contexts only at later stages. However, it was unclear whether these timing effects related to kinematic deployment or processing time. Here, we kept video duration constant across occlusion intervals, thus selectively manipulating the availability of kinematic information with a constant contextual processing time. Still, we showed reliable contextual effects across unfolding kinematics.

Crucially, occluding the action videos at later time intervals (i.e., 4 and 5) did not cancel out the contextual facilitation generated by exposure to high-probability scenarios. Indeed, although the unfolding kinematic cues became evident and the hand posture pointed at a specific action outcome, participants persisted in integrating contextual prior information in predict action intention. Their responses were more accurate when actions were embedded in contextual scenarios that were associated with a high vs. low probability of the observed action. These findings of a continuous “advantage” of prior context even in the presence of fully available kinematic information seem to contradict the view that prior context serves action understanding only when kinematic information is scarce and does not allow for disentangling action intention [9,23,24,25]. At these later stages of action unfolding, in spite of unequivocal action kinematics, the AON would still be impacted by the semantic representation of the action intention, as suggested by prior context. This suggests that context-based expectations are continuously integrated and compared with the upcoming kinematic information. If the two sources of information match, action understanding is facilitated; if the two sources of information clash, a prediction error is generated to update the prediction, hindering prompt action understanding. The evidence that action prediction performance progressively improved across the five intervals, being differently modulated by contextual expectations, crucially highlights the importance of timing during action observation, corroborating the view that the observer’s motor system may be sensible to different aspects of action representations during action deployment [40]. This suggests that action understanding is not exclusively promoted by the unconscious [9] or conscious [41,42] capture of specific kinematic cues. Contextual cues and kinematic information are continuously integrated during action observation to ensure prompt and efficacious prediction of the overarching action intention, independently of the availability of kinematics hints. Collectively, all these findings fit well with longstanding studies, highlighting the crucial role played by the probabilistic associative knowledge derived from previously encountered contextual information in supporting action recognition (e.g., [11,13,38,43,44]).

Concerning the second aim of the study, which was to test the existence of a possible correlation between subclinical autistic traits and the extent of contextual modulation on action prediction performance, our results unveiled a correlation between the attention-to-detail trait and the extent of contextual facilitation observed in the later occlusion point. No correlation was reliable at the initial occlusion interval, suggesting that all the participants, independently of their level of autistic traits, relied on prior context to predict action intention in the absence of reliable kinematic information. Conversely, we found that the higher the individual scores on the attention-to-detail subscale were, the higher the individual index of contextual facilitation at the final time interval was, where kinematics information was fully available. This is particularly remarkable in that it points to a dysfunctional integration between contextual and kinematic sources of information during action understanding in individuals with high levels of autistic traits.

Previous studies have provided evidence that action prediction performance in individuals with ASD is poorly supported by contextual information [19]. In Bayesian terms, ASD individuals experience deficits in the integration of actual sensory evidence with prior context, reflected in a polarized interplay between bottom-up and top-down processes in favor of the former [45]. In this regard, the Weak Central Coherence (WCC, [46]) and Enhanced Perceptual Functioning (EPF, [47]) hypotheses collectively share an over-reliance on sensory information, “clouding” the aid normally provided by contextual experience-dependent information. Further, it has been shown that the presence of subclinical autistic traits would also correlate with the lack of benefits associated with exposure to prior context [31]. Accordingly, there is consistent evidence considering autistic traits as a continuum across the general population [48], with ASD individuals lying at the extreme of this distribution; these traits can be reliably measured by means of the AQ [30] questionnaire.

It is of note that the observed correlation was specific to subclinical traits related to the attention-to-detail scale, which involves the perceptual propensity to focus on fine-grained aspects of sensory input at the expense of more integrated perceptions. A previous study demonstrated that during an audiovisual statistical learning paradigm, individuals with high scores on the attention-to-detail scale were likely to show reduced adaptations to the statistical temporal regularities of their environment [49]. Therefore, this subclinical trait seems particularly sensitive to the individual ability to generate an internal probability map based on prior sensorial experience [45].

Other studies, however, have failed to document a relation between contextual modulation of action prediction performance in condition of ambiguous kinematics and subclinical autistic traits at a behavioral level in the general population [18,20,21]. Differently, they reported an association between attention-to-detail scores and the inhibition of cortico-spinal facilitation in response to actions embedded in incongruent vs. congruent or ambiguous contexts [18] and a relationship between the extent of contextual modulation of observer’s corticospinal excitability during action prediction and individual levels of social skills [21]. This suggested an altered contextual modulation of the observer’s motor system that could be compensated for by higher-level processing of contextual information in individuals with subclinical autistic traits [18], but not in individuals with clinical ASD [19]. The findings of this study shed further light on this issue by showing an excessive, rather than a poor use of contextual information by individuals with high autistic traits in conditions of reliable kinematic information. This suggests that an atypicality of high autistic traits might be an altered weighting of contextual and kinematic cues according to their reliability, rather than a deficit in processing one or the other source of information. Our finding of augmented contextual facilitation in more meticulous individuals would suggest that contrary to views of decreased implicit learning in ASD [50], the learning of appropriate prior probabilities is not fully impaired.

However, since the present study was limited to neurotypical participants, this novel finding deserves more in-depth exploration in ASD to advance our understanding of the interplay between contextual and kinematic information in action prediction. We might speculate that in ASD and/or individuals with high levels of attention to detail, over-reliance on sensorial information becomes less prominent when it is highly reliable, and this allows these individuals to focus more on the details of contextual scenarios. Furthermore, our data call for further TMS studies probing the deployment of motor activation at different occlusion intervals to better describe motor resonance in relation to the interplay between evolving kinematic information and the strength of contextual prior.

In conclusion, although we acknowledge the intrinsic limitations of our behavioral study, which was limited to task performance measurements, our findings show clear evidence for the crucial role of context, which is continuously integrated with the sensory environment for guiding the understanding of actions. This consideration deserves further investigation not only considering the consistently reported deficits of contextual information integration in ASD [19] but also in addiction disorders, as in the case for environmental drinking context on social drinker’s ability to withhold responses to alcoholic cues [51]. The results of our study are relevant also for other clinical populations in which action observation is used for rehabilitation, as in patients with stroke [52], Parkinson’s disease [53], and cerebral palsy [54], possibly through modulation of the AON [55]. Our findings suggest that action observation training should involve actions that are not isolated but embedded into specific contexts. Furthermore, they should aim to reinforce the use of contextual information, for example, through performance feedback [56] or via non-invasive brain stimulation [57]. Of note, the reinforcement of expectations provided by context has been shown to be effective during virtual reality training, which aims to improve social cognition in patients with social perception difficulties due to cerebellar malformation [58].

## Figures and Tables

**Table 1:** Descriptive statistics of the AQ and subscale scores.

Subscales and Total	Mean	St. Dev	Range
Attention switching	4.5	1.7	0–9
Attention to detail	5	2.4	0–10
Communication	2.5	1.7	0–6
Imagination	2.6	1.7	0–8
Social skills	3	1.8	1–7
AQ total	17.6	5.7	6–29

**Table 2:** Contextual Facilitation Index of interval 1 and interval 5. The asterisk signals significant *p*-value.

Contextual Facilitation Index: Interval 1				
Coefficients	β	t	*p*-Level	Tolerance
Attention switching	−0.031	−0.211	0.833	0.661
Attention to detail	0.135	1.071	0.288	0.914
Communication	0.125	0.821	0.414	0.635
Imagination	−0.048	−0.382	0.703	0.946
Social skills	−0.08	−0.563	0.575	0.723
**Contextual Facilitation Index: Interval 5**				
**Coefficients**	**β**	**t**	***p*-Level**	**Tolerance**
Attention switching	0.212	1.517	0.134	0.661
Attention to detail	0.277 *	2.339	0.022	0.914
Communication	−0.1	−0.706	0.483	0.635
Imagination	−0.134	−1.148	0.255	0.946
Social skills	0.133	0.996	0.323	0.723

## Data Availability

The data presented in this study are openly available in OSF https://osf.io/2d3ph/?view_only=908e64ec6ae84a8482a5edfc69eaa4ec, accessed on 4 January 2024.
